# SpatialMap: Spatial Mapping of Unmeasured Gene Expression Profiles in Spatial Transcriptomic Data Using Generalized Linear Spatial Models

**DOI:** 10.3389/fgene.2022.893522

**Published:** 2022-05-26

**Authors:** Dalong Gao, Jin Ning, Gang Liu, Shiquan Sun, Xiaoqian Dang

**Affiliations:** ^1^ First Department of Orthopedics, The Second Affiliated Hospital of Xi’an Jiaotong University, Xi’an, China; ^2^ Sports Medicine and Joint Surgery, Xianyang Central Hospital, Xianyang, China; ^3^ School of Public Health, Xi’an Jiaotong University, Xi’an, China; ^4^ Key Laboratory of Trace Elements and Endemic Disease, Xi’an Jiaotong University, Xi’an, China

**Keywords:** scRNA-seq, spatial transcriptomics, generalized linear spatial model, integrative analysis, penalized quasi-likelihood

## Abstract

Recent advances in various single-cell RNA sequencing (scRNA-seq) technologies have enabled profiling the gene expression level with the whole transcriptome at a single-cell resolution. However, it lacks the spatial context of tissues. The image-based transcriptomics *in situ* studies (e.g., MERFISH and seqFISH) maintain the cell spatial context at individual cell levels but can only measure a limited number of genes or transcripts (up to roughly 1,000 genes). Therefore, integrating scRNA-seq data and image-based transcriptomics data can potentially gain the complementary benefits of both. Here, we develop a computational method, SpatialMap, to bridge the gap, which primarily facilitates spatial mapping of unmeasured gene profiles in spatial transcriptomic data *via* integrating with scRNA-seq data from the same tissue. SpatialMap directly models the count nature of spatial gene expression data through generalized linear spatial models, which accounts for the spatial correlation among spatial locations using conditional autoregressive (CAR) prior. With a newly developed computationally efficient penalized quasi-likelihood (PQL)-based algorithm, SpatialMap can scale up to performing large-scale spatial mapping analysis. Finally, we applied the SpatialMap to four publicly available tissue-paired studies (i.e., scRNA-seq studies and image-based transcriptomics studies). The results demonstrate that the proposed method can accurately predict unmeasured gene expression profiles across various spatial and scRNA-seq dataset pairs of different species and technologies.

## 1 Introduction

Single-cell RNA sequencing (scRNA-seq) is becoming an established experimental technique for transcriptome profiling to study the functional states of individual cells and to reveal all possible cell types/states (Tim and Satija, 2019; Ma et al., 2020; Lindeboom et al., 2021). Current scRNA-seq technologies/protocols allow whole-transcriptome sequencing in an unbiased manner (Picelli et al., 2014; Zheng et al., 2017; Mereu et al., 2020). However, these protocols commonly result in the dissociation of tissue of origin to single cells and therefore loss of spatial context. Since the isolated cells are out of their original tissue matrix or natural microenvironment, altered transcriptional states do not truly reflect the natural states of a cell within the tissue of origin (Ståhl Patrik et al., 2016; Burgess, 2019; Sun et al., 2020; Zhu et al., 2021), which is a crucial step toward understanding spatial heterogeneity of complex tissues (Antebi et al., 2017; Levy-Jurgenson et al., 2020; Saviano et al., 2020) ].

Single-molecule fluorescence *in situ* hybridization (smFISH) or image-based transcriptomics *in situ* studies (Battich et al., 2013) provide an unprecedented opportunity to decipher the functional states on the spatial landscape at single-cell or even subcellular resolution within the context of tissue of origin, such as MERFISH (Xia et al., 2019) and seqFISH (Shah et al., 2018). However, these protocols usually only assay a limited number of genes or transcripts and therefore biased transcriptome profiling of one particular cell. Although a recent evolution of sequential fluorescence *in situ* hybridization (e.g., seqFISH+) achieves super-resolution imaging and multiplexing of genes up to around 10,000 per cell (Linus Eng et al., 2019; Xia et al., 2019), it always involves the heavy computational burden with increasing data storage (Strell et al., 2019).

Overall, scRNA-seq technologies allow whole-transcriptome profiling but for the loss of spatial context, while image-based transcriptomics *in situ* studies maintain spatial context but profile a limited number of genes. Therefore, to bridge such a gap, an integrative analysis of scRNA-seq studies and image-based transcriptomics *in situ* studies is potentially able to gain complimentary benefits from both of them. Recently, many integrative methods were developed to map the single cells dissociated from scRNA-seq protocols to the spatial landscape of the same tissues of origin (James et al., 2014; Achim et al., 2015; Satija et al., 2015), while only one machine learning method, SpaGE, was developed for predicting the missing gene expression measurements in the spatial data *via* integrating with scRNA-seq data from the same tissue (Abdelaal et al., 2020). SpaGE integrates spatial and scRNA-seq datasets to predict unmeasured expression profiles in their spatial configuration. On the one hand, however, SpaGE requires the data transformation step of both data prior to performing the integrative analysis. However, it is documented in many other studies that analyzing normalized data can be suboptimal as this approach fails to account for the mean–variance relationship that existed in raw counts (Sun et al., 2017; Lun, 2018). On the other hand, SpaGE ignores the spatial correlation among spatial locations and therefore lost the spatial heterogeneity modeling in the analysis.

In this article, we develop SpatialMap, a robust statistical method to predict unmeasured gene expression profiles of each cell in spatial transcriptomic data through the integration with scRNA-seq data from the same tissue. SpatialMap relies on the generalized linear spatial models that directly model the count nature of gene expression levels of spatial transcriptomics data and accounts for the spatial heterogeneity using conditional autoregressive (CAR) prior (Julian and Kooperberg, 1995). Moreover, we develop a computationally efficient algorithm based on the penalized quasi-likelihood (PQL) algorithm (Sun et al., 2019). Finally, we apply SpatialMap on four tissue-paired datasets consisting of scRNA-seq data and image-based spatial transcriptomics data on the same tissue. The results demonstrate that SpatialMap can accurately predict unmeasured gene expression profiles across various spatial and scRNA-seq dataset pairs and would be, especially conducive to spatial downstream analysis when the image-based spatial data are poor in transcript profiles.

## 2 Results

### 2.1 SpatialMap Overview

**FIGURE 1:**
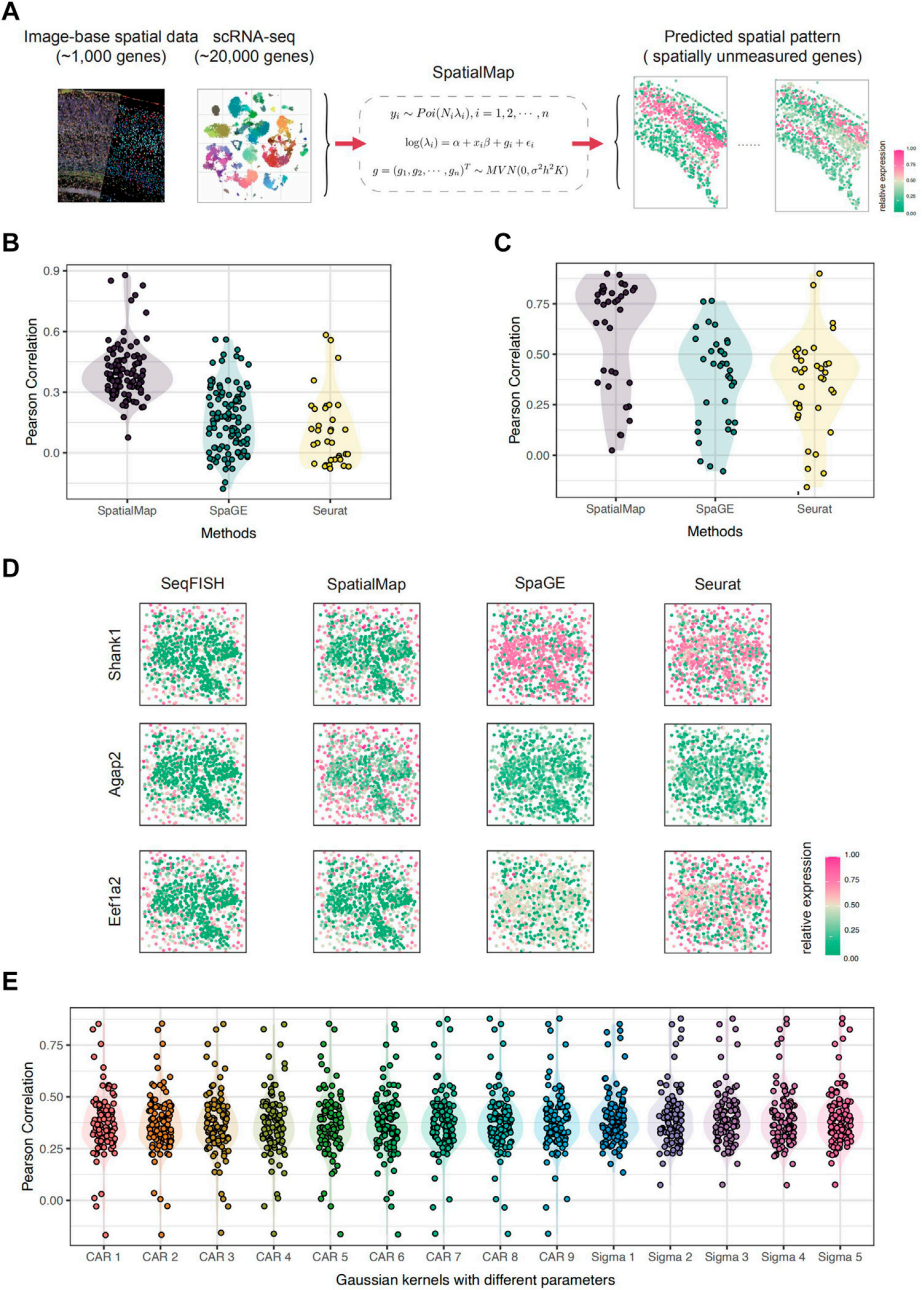
Overview of SpatialMap. **(A)** Pipeline of SpatialMap. SpatialMap takes tissue-paired image-based spatial data (contain about 1,000 genes) and scRNA-seq data (contain about 20,000 genes) as inputs, aiming to predict expression patterns for spatially unmeasured genes using a generalized linear spatial model. **(B)** Pearson correlations between predictions and original values of randomly selected 100 genes from experiments on the SeqFISH dataset. The mean values are 0.402, 0.168, and 0.108; minimum values are 0.074, −0.178, and −0.078; and maximum values are 0.878, 0.560, and 0.583 for SpatialMap, SpaGE, and Seurat, respectively. Each dot represents an individual gene. **(C)** Pearson correlations between predictions and original values of SPARK-selected 35 genes from experiments on the SeqFISH dataset. The mean values are 0.607, 0.377, and 0.355; minimum values are 0.024, −0.079, and −0.158; and maximum values are 0.899, 0.764, and 0.899 for SpatialMap, SpaGE, and Seurat, respectively. **(D)** Visualization of predicted spatial expressions. Pearson correlations are 0.825, 0.814, and 0.894 for SpatialMap-performed genes *Shank1*, *Agap2*, and *Eef1a2*, respectively. Color represents the relative gene-expression level (purple, high; green, low) **(E)** Pearson correlations between predictions and original values using different kernel functions in SpatialMap. “CAR 1” to “CAR 9” indicates CAR prior concerning spatial parameters being 0.1 to 0.9, and “Sigma 1” to “Sigma 5” indicates the Gaussian kernel with five chosen various scaling parameters. Color represents different kernels used in SpatialMap.

We developed a computational method, SpatialMap, to predict unmeasured gene expression levels of the spatial landscape using scRNA-seq data (Figure 1A). We briefly provide an overview of SpatialMap; more details are available in Methods and Materials. We take spatial and scRNA-seq gene expression matrices as inputs; to characterize spatially expressed patterns with genes, we examine one gene at a time. For each gene measured by image-based technologies, we model the raw read counts with a Poisson distribution, i.e.,

$$y_{i}∼Poi\left(N_{i}\lambda_{i}\right),\;i=1,2,\ldots ,n,$$ (1)

where *Poi*(⋅) is the Poisson distribution, *N_i_* is the summation of the total number of counts across all genes for spatial spot *i*, and *λ*_*i*_ is an unknown Poisson rate parameter, which can be modeled as the linear combination of the following terms:

$$\log\left(\lambda_{i}\right)=\alpha +x_{i}\beta +g_{i}+e_{i},$$ (2)

where *α* is an intercept; *x*_*i*_ refers to the cell-type-specific expression-level computed by scRNA-seq for the *i*th pixel, and *β* is its corresponding coefficient; *e*_*i*_ is the residual term that is independently and identically distributed following *N* (0, *σ* ^2^ (1 − *h* ^2^)), with *σ*_2_ and *h* ^2^ being the scaling factors; and *g*_*i*_ is a random effect modeling the spatial correlation pattern among spatial pixels with kernel functions, i.e.,

$$g={\left(g_{1},g_{2},\ldots ,g_{n}\right)}^{T}∼MVN\left(0,\sigma^{2}h^{2}K\right),$$ (3)

where *MVN* (⋅) denotes the multivariate normal distribution and the variance–covariance matrix *K* is a kernel function of the *n* spatial pixels. We set *h* ^2^ ∈ [0, 1], explaining the expression variance in log (*λ*_*i*_) owing to the random noise *g*. *e* is a *n*-vector following *MVN* (0, *σ* ^2^ (1 − *h* ^2^)*I*) with *I* being an *n* by *n* identity matrix that models an independent sequencing error. In addition, we also use conditional autoregressive (CAR) models as the prior distributions for spatial random effects with spatial data. By Brook’s Lemma, the joint distribution of spatial random effects can be modified as:

$$g∼MVN\left(0,\sigma^{2}h^{2}{\left(D-\alpha_{s}W\right)}^{-1}\right),$$ (4)

where *α*_*s*_ is a parameter that controls spatial dependence (*α*_*s*_ = 0 implies spatial independence and *α*_*s*_ = 1 collapses to an intrinsic conditional autoregressive (IAR) specification). *D* = diag (*m*_*i*_) is an *n* by *n* diagonal matrix with *m*_*i*_ = the number of neighbors for pixel *i*. *W* is the adjacency matrix (*w*_*ii*_ = 0, *w*_*ij*_ = 1 if *i* is a neighbor of *j*, and *w*_*ij*_ = 0 otherwise.

With the tissue-paired scRNA-seq data and spatial image-based data, we denote *x*_*i*_ as the average gene expression level across all cells within one particular cell type. The construction of generalized linear spatial models is regarded as the most crucial step. On the one hand, directly modeling on counts avoids information loss caused by the data normalization and scaling process. On the other hand, introducing random effects not only accommodate the widespread independent sequencing error but also explain nonindependent error among pixels due to the affinity in the physical space, fully carrying over-dispersion of sparse spatial transcriptome. Through parameter estimation and statistical inference, the spatial distribution of genes shared by scRNA-seq and spatial techniques can be figured out and served as references for predictions of genes without spatial resolution.

Cross-validation is used to display the power of SpatialMap in reproducing spatially expressed patterns. Specifically, genes shared by tissue-paired spatial and scRNA-seq datasets are used as ground truth. We remove one gene at a time and set the rest as the observed data to fit the generalized linear spatial model (GLSM). Here, for each removed gene, we choose its most correlated gene among the shared data, computed by their scRNA-seq expression profiles, as the reference gene, to predict the spatial patterns of the removed gene using estimates. Finally, correlations between predicted values and their ground truth are used for performance evaluation. We also perform prediction evaluation under different downsampling setups and kernel functions.

### 2.2 SpatialMap Outperforms SpaGE and Seurat on STARmap and seqFISH Datasets

We first applied SpatialMap on the tissue-paired seqFISH datasets (Linus Eng et al., 2019). There were 9,879 shared genes measured by both spatial and single-cell datasets, collected at 913 spatial pixels by the seqFISH technique in the cortex, subventricular zone, and choroid plexus of the mouse brain. For convenience, we only leverage 1,000 randomly selected genes for simulation experiments. We aim to attest excellent prediction performance of SpatialMap, compared with state-of-the-art methods, SpaGE and Seurat. We randomly select 100 genes and apply SpatialMap to the rest of the genes to figure out their spatial distribution for further prediction. SpaGE and Seurat are also performed based on the same settings, respectively. We evaluated the prediction performance by calculating the Pearson correlation between the originally measured spatially resolved genes and their corresponding prediction values. SpatialMap outperforms SpaGE and Seurat with a mean Pearson correlation at 0.388 (average at 0.388 with a standard error at 0.023), compared to a mean at 0.167 and 0.108 for SpaGE and Seurat, respectively (Figure 1B). Moreover, the best prediction for the *Syngap1* gene reaches a Pearson correlation at 0.878, using SpatialMap, and the highest correlations are 0.560 (*Sema6b*) and 0.582 (*Slain1*) (for SpaGE and Seurat, respectively).

We wonder whether having more measured spatial genes is always beneficial to predict the spatial patterns of unmeasured genes. To answer that, we take the number of known genes into account as sensitivity analysis. We fixed 100 genes as a test set and downsampled the remaining 100, 200, 300, 400, 500, and 600 genes to fit the model. However, the average Pearson correlations show only a slight change in prediction performance with a maximum at 0.3973 and a minimum at 0.3963. We check average correlations between the test set and their reference genes under different downsampling setups, and the results show similar minor changes, as the minimum average correlation is 0.1962 and the maximum average correlation is 0.2549, increasing with the number of known genes. Therefore, we may suspect that prediction performance is related to the correlation between unmeasured genes and their reference genes, rather than the number of known genes to fit the model.

Furthermore, since we are concerned with spatial gene-expression patterns, we test the prediction performance for spatially specific genes. SPARK is a method for detecting genes with spatial expression patterns in spatially resolved transcriptomics studies (Sun et al., 2020). SPARK directly models the count data generated from various spatially resolved transcriptomics techniques through generalized spatial linear models. We apply SPARK on the seqFISH dataset and select 35 significant (adjusted *p* − value < 0.05) spatially expressed genes. Prediction and correlation evaluation is performed, and the results indicate higher reproducibility of the spatially expressed genes (Figure 1C). The average Pearson correlation achieves 0.607, and the best result even reaches 0.898 (*Cnp*). We visually compared some highly correlated genes, *Shank1*, *Agap2*, and *Eef1a2* (Figure 1D). SpatialMap recovers their spatial patterns well, while SpaGE and Seurat failed to do so. They mistakenly predicted *Shank1* to be highly expressed across the tissue, while *Agap2* remains sparse everywhere.

**FIGURE 2:**
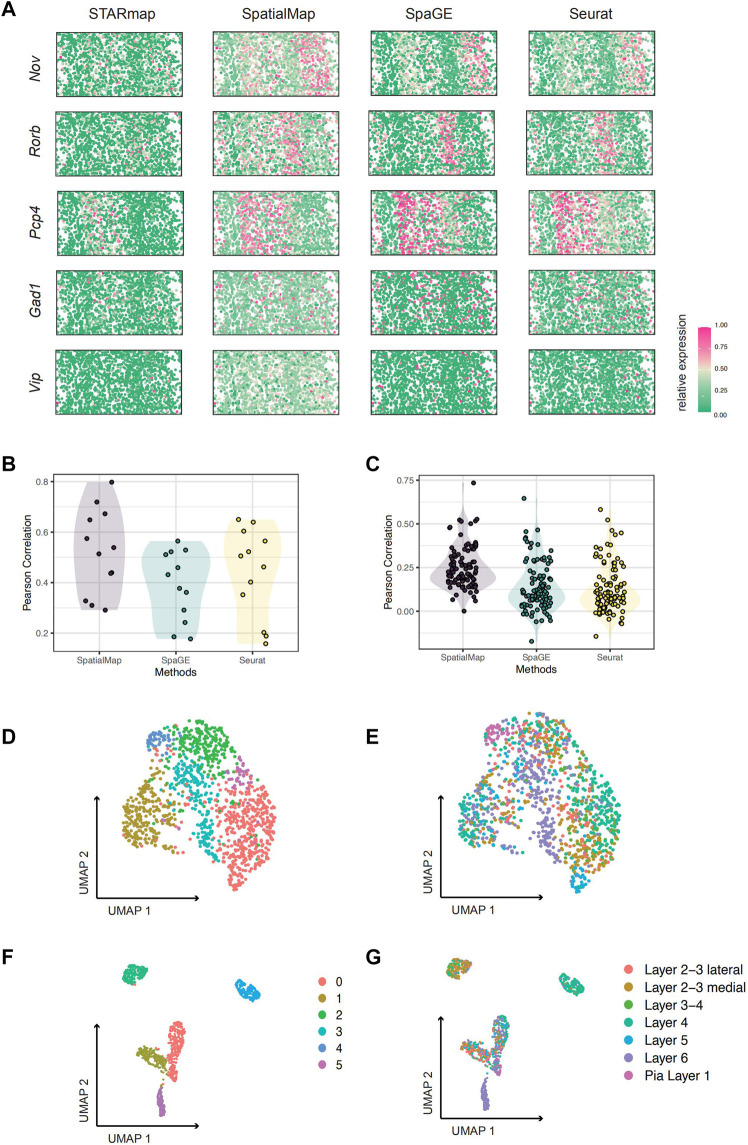
Performance evaluation on STARmap and osmFISH datasets. **(A)** Visualization of predicted spatial expressions. Pearson correlations are 0.320, 0.295, 0.518, 0.736, and 0.564 for SpatialMap-performed genes *Nov*, *Rorb*, *Pcp4*, *Gad1*, and *Vip*, respectively. **(B)** Pearson correlations between predictions and original values of 11 known marker genes from experiments on the STARmap dataset. The mean values are 0.522, 0.388, and 0.438; minimum values are 0.295, 0.177, and 0.158; and maximum values are 0.736, 0.565, and 0.650 for SpatialMap, SpaGE, and Seurat, respectively. **(C)** Pearson correlations between predictions and original values of randomly selected 100 genes from experiments on the STARmap dataset. The mean values are 0.250 (average at 0.194 with a standard error at 0.0235 among 10 repeat predictions), 0.142, and 0.129; minimum values are 0.0005, −0.172, and −0.144; and maximum values are 0.734, 0.646, and 0.582 for SpatialMap, SpaGE, and Seurat, respectively. **(D–G)** UMAP visualization of clustering performance on the osmFISH dataset. **(D,E)** Leiden clustering and UMAP based on original 33 genes from the osmFISH dataset, colored by cluster number **(D)** and cell type annotation labels **(E)**. **(F,G)** Leiden clustering and UMAP based on 43 genes by predictions using SpatialMap, colored by cluster number **(F)** and cell type annotation labels **(G)**. Dots represent individual spatial pixels.

In addition, we demonstrate the performance of SpatialMap about different kernels with parameters. The Gaussian kernel function with various scaling parameters and CAR prior with different spatial parameters are tested based on 100 genes (Figure 1E). Pearson correlations indicate that SpatialMap is stable to sort kernel functions and shows robustness in parameter selections. We next applied SpatialMap to the tissue-paired STARmap dataset (Wang et al., 2018). There are 994 shared genes, collected at 1,549 spatial pixels by the STARmap technique, in the mouse’s primary visual cortex. At first, we are interested in reproducing the spatial distribution of known cortical layer marker genes and interneurons. A total of 12 genes including seven excitatory neuron markers (*Slc17a1*, *Nov*, *Cux2*, *Rorb*, *Sulf2*, *Pcp4*, and *Ctgf*) and five inhibitory neuron markers (*Gad1*, *Pvalb*, *Sst*, *Npy*, and *Vip*) remained as a test set. We performed prediction on the rest of the 982 genes using SpaGE and Seurat to benchmark the performance of SpatialMap. We evaluated the prediction performance by calculating the Pearson correlation between the originally measured spatially resolved genes and their corresponding prediction values. The results indicate that SpatialMap outperforms SpaGE and Seurat on spatial pattern reproduction, with a mean Pearson correlation at 0.522, compared to 0.388 and 0.437 for SpaGE and Seurat, respectively (Figure 2B). To be more accurate, 100 spatially resolved genes are selected for testing prediction performance (Figure 2C). We performed using SpaGE and Seurat to benchmark SpatialMap; moreover, different kernel functions with various parameters for the covariance matrix in SpatialMap are tested as well. Pearson correlations demonstrate that SpatialMap works generally better than the other two methods and is fairly stable concerning different parameters.

We visualize spatially expressed patterns of predictions together with the original truth value for more intuition (Figure 2A). Only part of the genes, *Nov*, *Rorb*, *Pcp4*, *Gad1*, and *Vip* are visualized, with the Pearson correlations at 0.328, 0.291, 0.514, 0.719, and 0.575, respectively. Notably, *Gad1* and *Vip*, known markers of inhibitory neurons, displayed sparse spatial patterns but showed fairly high reductions using SpatialMap. Therefore, it may indicate the compatibility of SpatialMap to the sparse spatial transcriptome. As can be seen from the aforementioned simulations, although correlations between SpatialMap-predicted values and the original truth are low, it has quite improved power over SpaGE and Seurat and is sufficient to clearly show spatial patterns.

### 2.3 SpatialMap Accurately Predicts Unmeasured Gene Expression Profiles of OsmFISH and MERFISH Datasets

The OsmFISH (Codeluppi et al., 2018) dataset consists of only 33 marker genes of mouse somatosensory cortex tissue by the cyclic smFISH technique. The mouse somatosensory cortex was then divided into 11 specific regions by identifying the spatial-dominant cell types on a single section in previous studies (Codeluppi et al., 2018). Cross-validation on the OsmFISH dataset showing poor performance is one of the critical reasons for the missing association that is attributed to the small sample size (Wang et al., 2022). In the original dataset, Leiden clustering was performed on all 33 gene-expression profiles. However, visualization by UMAP (performed using five dimensions) does not clearly show distinct clusters (Figures 2D,E). Therefore, we consider improving the clustering based on predicting more spatially resolved gene patterns. After performing SpatialMap, UMAP visualization showed clear clusters (Figures 2F,G), consistent with the fact that two clusters far apart are also anatomically separate. Therefore, by predicting the spatial expression profiles, it is helpful to optimize the spatial clustering, which is conducive to the division of a data-driven spatial organization.

Next, based on the previous simulation experiments, SpatialMap has shown great predictive power in leave-*n*-genes-out cross-validations. However, all of these results were achieved using relatively small-scale spatial datasets, with SeqFISH, STARmap, and OsmFISH measured on just around 1,000 pixels. This raises the question of whether SpatialMap is capable of applications on larger-scale spatial datasets. To evaluate this performance, the MERFISH (Xia et al., 2019) spatial dataset together with its corresponding scRNA-seq dataset pair, collected from the preoptic region of the thalamus in mice, was used for simulations. The MERFISH dataset contains transcriptomics profiles of 160 genes measured on 4,975 pixels. Of these, 155 genes were selected in previous studies as the markers of different cell populations or associated with various neuronal functions. A total of 139 genes were shared, and four marker genes were selected for four main cell types, inhibitory neuron (*Gad1*), mature OD cells (*Mbp*), ependymal cells (*Cd24a*), and mural cells (*Myh11*). We apply SpatialMap to reproduce their spatial patterns and perform visualization compared with SpaGE and Seurat.

**FIGURE 3:**
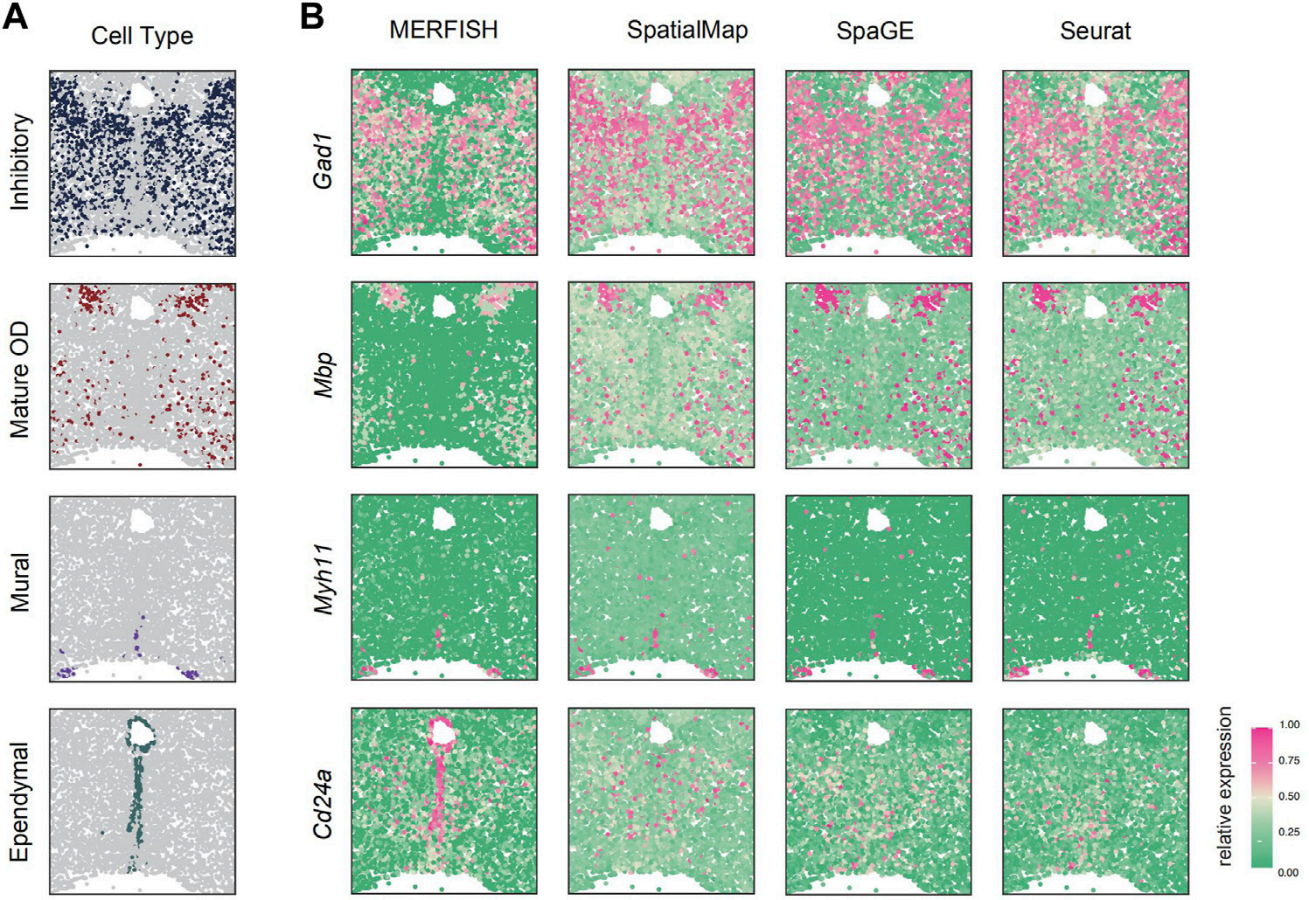
SpatialMap accurately predicted the expression of marker genes *Gad1*, *Mbp*, *Myhh11*, and *Cd24a*, consistent with MERFISH-measured data. **(A)** Spatial distribution of four main cell classes. The cell classes are represented by colored dots, while all other background cells are shown as gray dots. **(B)** SpatialMap-predicted genes (the second column) fairly recover their measured patterns by the MERFISH technique (the first column) and correspond to the distribution of cell types. Color represents the relative gene-expression level (purple, high; green, low).

The results indicated that SpatialMap could reproduce their spatially expressed patterns (Figure 3B) and is consistent with the distribution of cell types (Figure 3B). Compared with SpaGE and Seurat, SpatialMap shows clear spatial expression patterns and avoids overexpressed predictions, especially for the *Gad1* marker, while SpaGE and Seurat predicted a highly expressed pattern in the truly low-expressed regions. However, for the marker gene *Cd24a*, SpatialMap is equally unsatisfactory. It may be because of the low correlation (0.179) between the *Cd24a*-expression level and any other reference gene. Therefore, genes with distinct differences at scRNA-seq levels are not suitable as references for spatial pattern predictions.

## 3 Methods and Materials

### 3.1 SpatialMap: Model and Algorithm

We aim to model gene-expression data collected by various image-based spatial technologies, such as seqFISH, osmFISH, and MERFISH, using integration with single-cell RNA sequencing data referred to the same biological tissue. These spatial techniques simultaneously record gene-expression levels of *q* different genes on *n* different spatial sites on a tissue of interest, which we also refer to as spatial pixels. The gene-expression levels are usually obtained as counts for the fact that they are numbers of barcode mRNA for any given transcript in a single cell. The number of genes, *q*, often varies as many as ∼1,000, and the number of spatial sites randomly varies from a couple of hundreds to thousands. For the corresponding scRNA-seq data, the number of genes can achieve the whole transcriptome for every single cell.

The purpose to integrate image-based spatially resolved data with scRNA-seq collected on the same tissue is to predict spatially expressed patterns for those unmeasured genes. We map single-cell level expression patterns for cell types using a generalized linear spatial model (GLSM). GLSM is a generalized linear mixed model that directly models non-Gaussian spatial data and uses random effects to capture the underlying stationary spatial process (Christensen and Waagepetersen, 2002).

Specifically, SpatialMap uses two gene-expression matrices *Y*_*n*×*q*_ and *S*_*m*×*p*_ as inputs, corresponding to image-based spatial transcriptome data and scRNA-seq, respectively. We model one gene at a time. We denote *y*_*i*_, the *i*th row of *Y*_*n*×*q*_, as the gene-expression measurement in the form of counts for the *i*th spatial pixel. We denote *N*_*i*_ as the normalization factor for the *i*th pixel, computed as the summation of the total number of counts across all genes for the *i*th pixel. We consider modeling the observed *y*_*i*_ with an overdispersed Poisson distribution:

$$y_{i}∼Poi\left(N_{i}\lambda_{i}\right),\;i=1,2,\ldots ,n,$$ (5)

where *Poi* (⋅) is the Poisson distribution and *λ*_*i*_ is an unknown Poisson rate parameter, which can be modeled as the linear combination of the following terms:

$$\log\left(\lambda_{i}\right)=\alpha +x_{i}\beta +g_{i}+e_{i},$$ (6)

$$g={\left(g_{1},g_{2},\ldots ,g_{n}\right)}^{T}∼MVN\left(0,\sigma^{2}h^{2}K\right),$$ (7)

$$e={\left(e_{1},e_{2},\ldots ,e_{n}\right)}^{T}∼MVN\left(0,\sigma^{2}\left(1-h^{2}\right)I\right),$$ (8)

where *α* is an intercept; *x*_*i*_ refers to the expression-level of the cell type mapped to the *i*th pixel; *β* is the corresponding coefficient; *e*_*i*_ is the residual error that is independently and identically distributed following *N* (0, *σ* ^2^ (1 − *h* ^2^)), with *σ*_2_ and *h* ^2^ being the scaling factors; and *g*_*i*_ is a random effect modeling the spatial correlation pattern among spatial pixels, where *MVN* (⋅) denotes the multivariate normal distribution and the variance–covariance matrix and *K* is a kernel function of the *n* spatial pixels using Gaussian kernel or CAR prior. We set *h* ^2^ ∈ [0, 1], explaining the expression variance in log (*λ*_*i*_) owing to the random noise *g*. *e* follows a multivariate normal distribution, and *I* is a *n* by *n* identity matrix.

For image-based spatial data, each count represents a gene-expression measurement in a single cell at that pixel. Therefore, finding an embedded mapping between single cells and spatial pixels can directly make the integration work. However, due to inconsistency in the number of cells and number of pixels, mapping between cell types and spatial pixels is constructed as a replacement due to the following considerations: first, tissue dissociation before scRNA-seq is unlikely to preserve all the cells, resulting in information imbalance between single-cell and spatial techniques. In addition, spatial data and scRNA-seq data are often collected from two different experiments, hardly retaining one-to-one correspondence among pixels and cells. Moreover, the spatial characteristics of transcriptomes and their spontaneous impacts are the research purpose instead of the precise physical location of each cell in a tissue. Therefore, we set the gene-expression level for each cell type as the average of cells within that type, simply referred to as the center. The center is mapped to its corresponding pixels based on Pearson correlation in the expression space. Cell type identification can be based on previous annotations or other clustering methods.

We evaluated the prediction performance using cross-validation. *N* genes shared by spatial and scRNA-seq datasets are used as ground truth; *n* genes are left out, and the remaining *N* − *n* genes are used for integration and spatial mapping of *n* “unmeasured” expression profiles in spatial data. The prediction is then evaluated by comparing the original measurement and the predicted expression profiles of the removed genes. We have two kinds of gene left-out experiments. On the one hand, we intend to randomly select some genes and evaluate the prediction performance of all the methods to draw their ability in general. On the other hand, considering that we are interested in genes that display spatial patterns rather than their exact gene expression level, we identify some genes with spatial expression patterns (referred to as SE genes) and perform prediction evaluations, to characterize the performance of all the methods on them.

### 3.2 Algorithms

In GLSM, whether the single-cell gene-expression level that is helpful to characterize its spatial pattern conducive to the selected genes which serve well as a reference to predict spatial patterns for more unmeasured ones is tested. Also, it can be translated into testing the null hypothesis “*H*_0_: *β* = 0.” Parameter estimation and hypothesis testing in GLSM is notoriously difficult, as the GLSM likelihood consists of an *n*-dimensional integral that cannot be solved analytically. Therefore, we develop an approximate-inference algorithm based on the penalized quasi-likelihood (PQL) approach (Lin and Norman, 1996; Sun et al., 2019). The PQL approach employs an iterative numerical optimization procedure. In each iteration, we introduce a set of continuous pseudodata $\hat{y}$ to replace the originally observed count data *y*. The pseudodata $\hat{y}$ is obtained based on a second-order Taylor expansion using the conditional distribution *P* (*y*_*i*_|*β*, *e*) with the first- and second-order moments *E* (*y*_*i*_|*β*, *e*) and *V* (*y*_*i*_|*β*, *e*), both evaluated at the current estimates of the fixed coefficients *β* as well as the nugget effects *e*. With the pseudodata, the complex GLSM likelihood function for the original data *y* is replaced by a much simpler LMM likelihood function for the pseudodata $\hat{y}$ , thereby alleviating much of the computational burden associated with GLSM. With the pseudodata $\hat{y}$ , we can perform inference and update parameters using the standard average information (AI) algorithm for LMMs (Yang et al., 2011; Han et al., 2016). By iterating between the approximation step of obtaining the pseudodata $\hat{y}$ and the inference step of updating the parameter estimates *via* the AI algorithm, the PQL approach allows us to perform inference in a computationally efficient fashion. To further improve the computational speed, we also take advantage of the parallel computing environment readily available in modern desktop computers nowadays and implement our method with multiple-thread computing capability using Rcpp.

The statistical power of such a hypothesis test will inevitably depend on how well the spatial kernel function *K* matches the true underlying spatial patterns. Therefore, the Gaussian kernel with different scaling parameters and kernel from the conditional auto-regression (CAR) model (Duncan et al., 2014) with different spatial parameters are performed in experiments to show quite a robustness of SpatialMap. With parameter estimates from the PQL-based approach, we compute the *p* − value and select genes with *p* − value <0.05 as a reference set. For those spatially unmeasured genes, we compute their Pearson correlation with the reference set in the single-cell expression space and extract the most “correlated” gene with its estimates $\hat{\Theta }=\left\{\hat{\alpha },\hat{\beta },{\hat{\sigma }}^{2},{\hat{h}}^{2}\right\}$ ; $\log\left({\hat{\lambda }}_{i}\right)=\hat{\alpha }+X_{i}^{\left(un\right)}\hat{\beta }+{\hat{g}}_{i}+{\hat{\epsilon }}_{i}$ is computed with *X* ^(*un*)^ being valued of that spatially unmeasured gene, and its predicted spatial pattern can be finally sampled.

### 3.3 Datasets Used in This Study

We used four image-based spatial datasets (SeqFISH (Linus Eng et al., 2019), STARmap (Wang et al., 2018), OsmFISH (Codeluppi et al., 2018), and MERFISH (Xia et al., 2019)) and two scRNA-seq datasets (AllenVISp (Tasic et al., 2018) and Moffit (Moffitt Jeffrey et al., 2018)). For image-based data, the SeqFISH dataset contains 10,000-gene probes to image 913 cells in the cortex, subventricular zone (SVZ), and choroid plexus of the mouse brain using the SeqFISH+ technique. It can be downloaded at https://github.com/CaiGroup/seqFISH-PLUS. The STARmap dataset contains 1,020 genes on 1,549 cells using the STARmap technique of the mouse primary visual cortex. The corresponding data and preprocessing code can be downloaded at https://github.com/weallen/STARmap. The OsmFISH dataset contains 33 genes of 1,290 cells collected from the mouse somatosensory cortex using cyclic smFISH. It can be downloaded at http://linnarssonlab.org/osmFISH/. The MERFISH dataset is collected on the mouse preoptic region of the hypothalamus from Dryad. We used the slice at Bregma +0.11 mm from animal 18 for analysis, as it contains 155 genes measured on 4,975 cells. It can be downloaded at https://datadryad.org/stash/dataset/doi:10.5061/dryad.8t8s248. The downloaded data have already been normalized. To obtain the raw count data, we rescaled the expression values by first multiplying by 1,000, adjusting for cell volume, and then converting the rescaled value into integers by taking the ceiling over the rescaled data. For scRNA-seq data, the AllenVISp (GSE115746) dataset contains scRNA-seq of 12,714 cells from the mouse primary visual cortex (VISp) with cell-type annotations. The Moffit (GSE113576) dataset contains scRNA-seq of ∼ 31,000 cells dissociated and captured from the preoptic region of the hypothalamus from multiple male and female mice.

During preprocessing of AllenVISp data, cells with the read depth (computed by the summation of the read counts across genes) less than 10 were removed, and cells labeled as “No class” or “Low Quality” are also left out according to the known metadata. During preprocessing of Moffit data, cells with the read depth less than 100 or labeled as “Ambiguous” or “Unstable” were removed. Cell filtration keeps the same for all the three methods. SpaGE and Seurat require data normalization (log-transformation using scaling factor 1e4), while SpatialMap takes raw count data as input. Other details on preprocessing of four spatial datasets are described in the Results part.

## 4 Discussion

We develop a computational method, SpatialMap, based on the generalized linear mixed model for mapping scRNA-seq into the spatial landscape. The predictive performance of SpatialMap is highly dependent on the construction and inference of statistical models for spatial gene expression patterns. In a statistical sense, the spatial distribution characteristics of each gene are outlined more directly and clearly, providing a reliable distribution rule for the prediction of the spatial pattern of new genes. In the process of data preprocessing, SpatialMap only needs to filter the pixels or genes with low expression and does not need to normalize or scale the data, to greatly avoid the interference and ambiguity of the latent information during the classical data preprocessing.

By simulations, we demonstrate that SpatialMap can effectively restore the spatial patterns of genes with significant spatial expression patterns. Compared with the present methods, SpatialMap can better display the relative gradient of gene distribution. For extremely sparse genes, SpatialMap can also capture their distribution to some extent. However, genes with highly sparse measurements in the downstream analysis generally do not provide significant information. Thus, it is recommended to be filtered in the preprocessing step. Comparing the prediction performance in SeqFISH data under the cross-validation set up of the randomly-selected genes and SPARK-selected-SE genes, SpatialMap managed to achieve higher correlations in genes with spatial patterns. We suspect that there are two reasons: on the one hand, SPARK can identify only 35 genes as SE genes, so the high correlations of some individual genes lead to a higher average level; on the other hand, we fit the models using genes with both spatial measurements and scRNA-seq expression profiles, among which the most correlated one in the scRNA-seq expression space is selected as the reference to predict spatially unmeasured genes. The average correlation level between the 100 randomly selected genes and their reference genes in scRNA-seq is 0.206 (with a minimum at 0.068 and a maximum at 0.674), while the average correlation level between the 35 SE genes selected by SPARK and their reference genes in scRNA-seq is 0.350 (with a minimum at 0.022 and a maximum at 0.616). Therefore, measured in the scRNA-seq expression space, the more dissimilar the reference gene is to the gene to be predicted, the worse the predictive performance will be.

SpatialMap can directly capture data characteristics. Based on the relationship between the expression of genes at the single-cell resolution and the configuration of spatial patterns, SpatialMap can fit the spatial distributions of genes well by establishing adaptive distribution and is supported by a powerful parameter estimation algorithm, which greatly improves the application and efficiency of the method.

## Data Availability

All source code used in our experiments and the processed data has been deposited at https://sqsun.github.io/software.html.
